# Cancellation of Auxetic Properties in F.C.C. Hard Sphere Crystals by Hybrid Layer-Channel Nanoinclusions Filled by Hard Spheres of Another Diameter

**DOI:** 10.3390/ma14113008

**Published:** 2021-06-01

**Authors:** Jakub W. Narojczyk, Krzysztof W. Wojciechowski, Jerzy Smardzewski, Attila R. Imre, Joseph N. Grima, Mikołaj Bilski

**Affiliations:** 1 Institute of Molecular Physics, Polish Academy of Sciences, M. Smoluchowskiego 17, 60-179 Poznań, Poland; narojczyk@ifmpan.poznan.pl; 2 Akademia Kaliska im. Prezydenta Stanisława Wojciechowskiego, Nowy Świat 4, 62-800 Kalisz, Poland; 3 Department of Furniture Design, Faculty of Wood Technology, Poznań University of Life Sciences, Wojska Polskiego 38/42, 60-627 Poznań, Poland; jsmardzewski@up.poznan.pl; 4 Department of Energy Engineering, Faculty of Mechanical Engineering, Budapest University of Technology and Economics, Muegyetem rkp. 3, H-1111 Budapest, Hungary; imreattila@energia.bme.hu; 5 Centre for Energy Research, Department of Thermohydraulics, POB. 49, H-1525 Budapest, Hungary; 6 Department of Chemistry, Faculty of Science, University of Malta, MSD 2080 Msida, Malta; joseph.grima@um.edu.mt; 7 Metamaterials Unit, Faculty of Science, University of Malta, MSD 2080 Msida, Malta; 8 Institute of Applied Mechanics, Poznań University of Technology, Jana Pawla II 24, 60-965 Poznań, Poland; mikolaj.bilski@put.poznan.pl

**Keywords:** auxetics, negative Poisson’s ratio, nanolayers, nanochannels, hard spheres, inclusions, Monte Carlo simulations

## Abstract

The elastic properties of f.c.c. hard sphere crystals with periodic arrays of nanoinclusions filled by hard spheres of another diameter are the subject of this paper. It has been shown that a simple modification of the model structure is sufficient to cause very significant changes in its elastic properties. The use of inclusions in the form of joined (mutually orthogonal) layers and channels showed that the resulting tetragonal system exhibited a complete lack of auxetic properties when the inclusion spheres reached sufficiently large diameter. Moreover, it was very surprising that this hybrid inclusion, which can completely eliminate auxeticity, was composed of components that, alone, in these conditions, *enhanced* the auxeticity either slightly (layer) or strongly (channel). The study was performed with computer simulations using the Monte Carlo method in the isothermal-isobaric (NpT) ensemble with a variable box shape.

## 1. Introduction

Auxetic materials [1] play an important role in designing and developing modern metamaterials. The word “auxetics” refers to materials for which the Poisson’s ratio (PR) [2] takes negative values [3]. Originating from the first theoretical models exhibiting negative Poisson’s ratio [4,5,6,7], through the construction of model materials with arbitrarily prescribed properties [8], up to ideas more commonly related today to auxetics [9,10,11], these relatively recently discovered materials became the subject of intense studies in the last few decades. Their extraordinary properties were quickly noted by the scientific community, what has been reflected in the rapidly growing knowledge base [12,13]. Auxetic properties were found not only in metamaterials [14,15,16] or model structures [17,18], but also in crystals, e.g., cubic systems [19], both theoretically [20,21,22,23] and experimentally [24,25]. Novel structures [26,27,28,29,30,31,32,33,34], nanostructures [35,36], and models [37,38,39,40,41,42,43,44], real materials (e.g., polymers [45,46], composites [47], or foams [48,49]), and metamaterials [50,51] with auxetic properties have been designed and reported. These advances in real metamaterials would not have been possible without more basic research [52,53,54], analysis [55,56,57,58,59], and optimization [60,61] of auxetic models on various levels. This effort is motivated by the extraordinary, counterintuitive elastic properties and potential applications of auxetics [62,63,64,65,66,67,68].

One of directions in the optimization of the properties of metamaterials is the study of inclusions in structures [69,70,71,72]. Recently, studies on elastic properties of particle model systems with inclusions of particles with other dimensions have been published. The inclusions in the form of channel [71] or layer [72] arrays have been introduced into f.c.c. crystals of hard spheres. The hard sphere (HS) potential constitutes a fundamental theoretical model, used for more than a half of century, to describe qualitatively the properties of condensed matter systems, such as solids and liquids [73]. This is due to the fact that short-range intermolecular correlations are well reproduced by the hard interactions. Thus, because of the fact that only purely geometrical interactions had been considered, the studies aimed to answer the question how the change of the size of inclusion particles influences the model’s elastic properties, with particular emphasis on Poisson’s ratio. It has been shown that (in the same thermodynamic conditions) both types of inclusions significantly impact the symmetry of the system. In both cases, this resulted in systems that belong to the tetragonal 422 symmetry class [74]. However, the influence of both of these inclusions on the elastic properties were notably different. Namely, the introduction of an array of channels of hard spheres led to an essential enhancement of auxetic properties. An increase in the number of crystallographic directions with negative Poisson’s ratio (i.e., $\left[111\right]\left[11\bar{2}\right]$, in which ν decreased from 0.065 down to −0.365) was accompanied by a decrease of the minimal values of Poisson’s ratio (in some directions, even down to −0.873) [71]. On the other hand, the use of a periodic stack of layer inclusions resulted in a small enhancement of the auxetic properties (when compared to a cubic system). Thus, one might expect that an array of hybrid inclusions, each formed by a layer and a channel oriented perpendicularly to each other, will strongly enhance the auxeticity in the system. The present paper is devoted to the verification of this hypothesis.

The paper is organized as follows: The most important aspects of the studied model are described in the following section. In Section 3, the derivation of the elastic properties in the NpT ensemble is briefly described, and the details of computer simulations are provided. The results of the study and their discussion are given in Section 4, whereas the last section (Section 5) contains a summary and conclusions.

## 2. The Model

**Figure 1:**
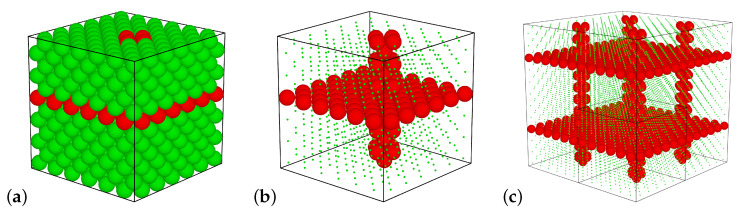
The unit supercell (f.c.c.) with the matrix and inclusion particles marked, respectively, in green and in red color (**a**). The inclusion’s structure (**b**) and the repeated supercell (**c**). The diameters of the matrix spheres in (**b**,**c**) were scaled down to show the underlying structure.

We considered the model of *N* spheres interacting with a pair potential of the form:

(1) $$\beta u_{ij}=\left\{\begin{array}{cc} \infty , & r_{ij}<\sigma_{ij},\\ 0, & r_{ij}\geq \sigma_{ij}. \end{array}\right.$$

where $r_{ij}$ is the distance between the centers of the interacting spheres *i* and *j*, $\sigma_{ij}=\left(\sigma_{i}+\sigma_{j}\right)/2$, $\beta =1/(k_{B}T)$, with $\sigma_{i}$, $\sigma_{j}$ being the sphere diameters, $k_{B}$ the Boltzmann constant, and *T* the temperature in Kelvins. Initially, all spheres in the system had a diameter equal to σ (which constitutes the unit of length) and formed an f.c.c. lattice. Such a system was then modified by the selection of $N_{\mathrm{inc}}=\left(5+2N_{x}\right)N_{y}-1$ spheres and replacing them with spheres with different diameters $\sigma^{\prime }\neq \sigma$. Amongst the $N_{\mathrm{inc}}$ spheres, $2N_{x}N_{y}$ lied in the selected crystallographic layer, orthogonal to the [001]-direction (as in [72]), whereas the remaining $5N_{y}$ spheres formed a channel with the axis parallel to the [001]-direction (as in [71]). Values $N_{x}$ and $N_{y}$ are the number of f.c.c. unit cells in the respective directions. The other $N-N_{\mathrm{inc}}$ spheres formed the so-called matrix for the inclusion spheres. Thus, after this modification, the considered system can be seen as periodic repetitions of a single supercell (Figure 1a,b), which form a stack of nanolayers (infinite in the xy-plane) joined with an array of nanochannels (Figure 1c). In the remaining part of the article, we refer to this modification simply as the inclusion.

The model was studied under periodic boundary conditions. The obtained results for the periodic box containing the single supercell were compared with simulations of periodic box containing systems: doubled in one selected *x*-, *y*-, or *z*-direction (doubled supercell), doubled in two selected directions (quadrupled supercell), and doubled in all three directions (octupled supercell) [72]. As the results agreed within the limit of experimental error, it was meaningful to simulate single supercells.

When gradually changing the value of $\sigma^{\prime }/\sigma$ (decreasing or increasing) from unity, one obtains a system with tetragonal symmetry (which will be discussed in the Results Section). It should be stressed that in this work, we limited our research to systems exhibiting tetragonal symmetry.

## 3. Method

The elastic properties of the studied systems were determined by computer simulations using the idea of Parrinello–Rahman [73,75,76]. Shape fluctuations of the periodic box were applied to the Monte Carlo method in the isobaric–isothermal ensemble (NpT). The following subsection describes how to calculate the elastic properties for crystals of arbitrary symmetry. The details regarding the simulation parameters are given in Section 3.2.

### 3.1. Theory

The Parrinello–Rahman method allows one to perform calculations of the elastic compliance tensor elements $S_{\alpha \beta \gamma \delta }$ of the considered model. During the simulation, the periodic box containing the system was allowed to change its parallelepiped shape [73,75,76]. The mentioned elements were obtained directly from these shape fluctuations. If one considers the periodic box to be described by a symmetric matrix h (formed by vectors defining the edges of the box or, more formally, a periodic parallelepiped), the strain tensor ε can be obtained from the fluctuations of h elements as [73,76]:

(2) $$\varepsilon =\frac{1}{2}\left(h_{p}^{-1}.h.h.h_{p}^{-1}-I\right)\;,$$

where I is a unit matrix and $h_{p}$ is the reference matrix, i.e., the average value of the h matrix at equilibrium under the dimensionless pressure $p^{*}=p\beta \sigma^{3}$, $h_{p}\equiv <h>$. To relate the elastic compliance tensor elements with the strain tensor components, the following formula was used [73]:

(3) $$S_{\alpha \beta \gamma \delta }=\beta V_{p}\langle \Delta \varepsilon_{\alpha \beta }\Delta \varepsilon_{\gamma \delta }\rangle \;,$$

where $V_{p}=\left|\det\left(h_{p}\right)\right|$ is the volume of the system at the dimensionless pressure $p^{*}$, $\Delta \varepsilon_{\alpha \beta }=\varepsilon_{\alpha \beta }-\langle \varepsilon_{\alpha \beta }\rangle$, $\langle \varepsilon_{\alpha \beta }\rangle$ is the average in the NpT ensemble, and α,β,γ,δ = *x*, *y*, or *z*.

An expression for Poisson’s ratio in relation to the elastic compliance tensor can be given in a general from [77]:

(4) $$\nu_{nm}=-\frac{m_{\alpha }m_{\beta }S_{\alpha \beta \gamma \delta }n_{\gamma }n_{\delta }}{n_{\zeta }n_{\eta }S_{\zeta \eta \kappa \lambda }n_{\kappa }n_{\lambda }}\;.$$

It follows from Equation (4) that Poisson’s ratio depends on the choice of two mutually orthogonal directions: the direction of the applied external stress (represented by the $\vec{n}$ vector) and the direction in which Poisson’s ratio is measured ($\vec{m}$). Both are unit vectors. The graphical example of these directions and their relations is presented in Figure 2 in [72]. In Equation (4), the *n*, *m* indices correspond to the respective vectors with $n_{\alpha }$, $m_{\beta }$ being their respective components. One should also note that the Einstein summation convention was used on Greek indices. For the sake of clarity, we replaced the $S_{\alpha \beta \gamma \delta }$ tensor with the elastic compliance matrix S (a symmetric square matrix of dimension six) using the Voigt representation [74]. The Latin indices for $S_{ij}$ elements took the values i,j=1,…,6.

It should also be stressed that all calculations in this work were for infinitesimally small strains. To study a case of large deformations, one would require the method described in [4]. Such a case was outside the scope of this research and will be the subject of future studies.

### 3.2. Simulations

The research was performed by the computer simulations using the Monte Carlo (MC) method in the NpT ensemble. The size of the considered supercell matched 6×6×6 f.c.c. cells, thus containing N=864 spheres, $N_{\mathrm{inc}}=101$ of which formed the inclusion. The systems where subjected to the dimensionless pressure $p^{*}=100,250,$ and 1250, as well as the $\sigma^{\prime }/\sigma$ values from the range between 0.95 and (depending on the pressure) 1.055. Ten independent simulation runs were performed for each value of $\sigma^{\prime }/\sigma$ and $p^{*}$. Each simulation took at least $10^{7}$ MC cycles, from which the first $10^{6}$ were rejected as the period in which the system reached thermodynamic equilibrium. The remaining details of the computer simulations can be found in [71,72].

## 4. Results and Discussion

**Figure 2:**
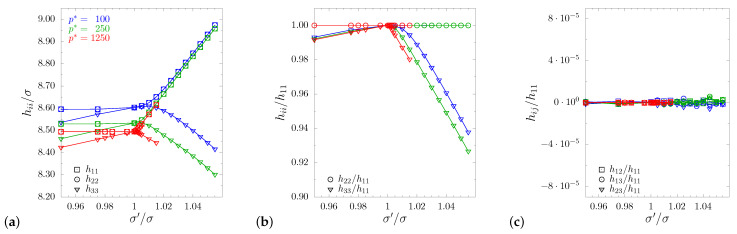
Box matrix elements for all studied values of dimensionless pressure (indicated in different colors). Diagonal components are presented in (**a**) and their ratios in (**b**), whereas the off-diagonal components (with relation to $h_{11}$) are presented in (**c**), which shows that in all cases, one obtains a cuboid box.

The research results obtained in our previous studies for systems with channels [71] and layers [72], studied at dimensionless pressure $p^{*}=100$, constitute a reference point for the present discussion. This enables one to assess the impact of hybrid inclusion on elastic properties of the crystal, compared to the effects exerted by each of the individual components alone. In the current work, apart from the comparison to previous models, an analysis of different pressure values, as a second parameter impacting the elastic properties of the sphere system with hybrid inclusions, has been also provided. As one might expect, the introduction of the inclusions forced the change of the systems’ symmetry from cubic ($\sigma^{\prime }/\sigma =1$) to tetragonal ($\sigma^{\prime }/\sigma \neq 1$). In Figure 2a–c, the changes of the system’s periodic box are shown. The figures present elements of box matrix h with respect to $\sigma^{\prime }/\sigma$ for all studied pressures. In all cases, at equilibrium, the matrix $h_{p}$ took the form [71,72,74]:

(5) $$h_{p}\equiv \langle h\rangle =\left[\begin{array}{ccc} \langle h_{11}\rangle & 0 & 0\\ 0 & \langle h_{11}\rangle & 0\\ 0 & 0 & \langle h_{33}\rangle \end{array}\right]\;,$$

**Figure 3:**
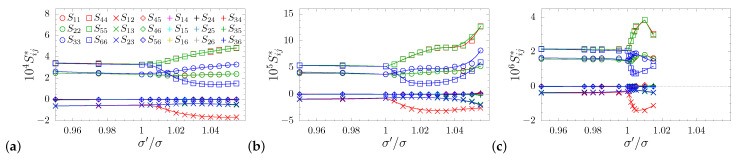
The dimensionless elastic compliance matrix elements ($S_{ij}^{*}=k_{B}TS_{ij}/\sigma^{3}$) for different dimensionless pressure values studied in this work: $p^{*}=$ (**a**) 100, (**b**) 250, and (**c**) 1250.

In Figure 3, which presents the elements of elastic compliance matrix S, one can also observe that within the studied range of $\sigma^{\prime }/\sigma \neq 1$ for all pressures, the symmetry was always tetragonal. In all cases, the relations among the $S_{ij}$ elements, characteristic of the tetragonal, 422 symmetry class [74], were preserved, namely: $S_{11}=S_{22}$, $S_{44}=S_{55}$, and $S_{13}=S_{23}$, as well as $S_{ij}=0$ for: i=1,…,5, j=4,5,6, i≠j. Thus, compliance matrix took the form:

(6) $$S=\left[\begin{array}{cccccc} S_{11} & S_{12} & S_{13} & 0 & 0 & 0\\ \cdot & S_{11} & S_{13} & 0 & 0 & 0\\ \cdot & \cdot & S_{33} & 0 & 0 & 0\\ \cdot & \cdot & \cdot & S_{44} & 0 & 0\\ \cdot & \cdot & \cdot & \cdot & S_{44} & 0\\ \cdot & \cdot & \cdot & \cdot & \cdot & S_{66} \end{array}\right]\;.$$

In this article, we restricted our discussion only to these values of $\sigma^{\prime }/\sigma$ (different at different pressures), for which the system maintained tetragonal symmetry, i.e., up to the values for which the elements of the elastic compliance matrix $S_{ij}$ with indices 3<i,j≤6 and i<j remained zero (see Figure 3).

The matrix S is in direct relation with the matrix of elastic constants B by the following tensor equality [73,78]:

(7) $$S_{iklm}B_{lmpq}=\frac{1}{2}\left(\delta_{ip}\delta_{kq}+\delta_{iq}\delta_{kp}\right)\;$$

**Figure 4:**
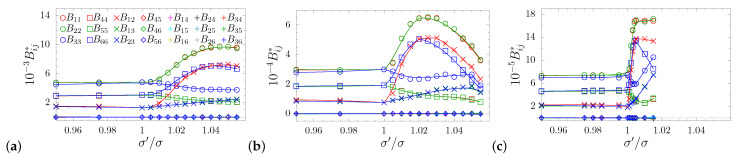
The dimensionless elastic moduli matrix elements ($B_{ij}^{*}=\beta B_{ij}\sigma^{3}$) for different dimensionless pressure values studied in this work: $p^{*}=$ (**a**) 100, (**b**) 250, and (**c**) 1250.

Thus, it is also a symmetric matrix of the form:

(8) $$B=\left[\begin{array}{cccccc} B_{11} & B_{12} & B_{13} & 0 & 0 & 0\\ \cdot & B_{11} & B_{13} & 0 & 0 & 0\\ \cdot & \cdot & B_{33} & 0 & 0 & 0\\ \cdot & \cdot & \cdot & B_{44} & 0 & 0\\ \cdot & \cdot & \cdot & \cdot & B_{44} & 0\\ \cdot & \cdot & \cdot & \cdot & \cdot & B_{66} \end{array}\right]\;,$$

which is clearly reflected in the data shown in Figure 4.

**Figure 5:**
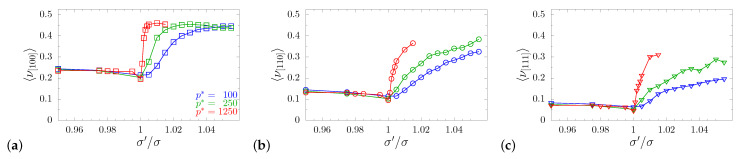
Poisson’s ratio averaged over all $\vec{m}$ directions, with external stress applied in selected $\vec{n}$ directions: (**a**) [100], (**b**) [110], and (**c**) [111].

Using Equation (4), $\nu_{nm}=-S_{nnmm}/S_{nnnn}$, and the knowledge of the S matrix, one can derive the values of Poisson’s ratio for any pair of mutually orthogonal directions $\vec{n}$ i $\vec{m}$. The equation holds for any crystalline symmetry, but, as mentioned before, in this work, we restricted our study to the range of tetragonal symmetry only. The reason was to show the influence of the metamaterial’s structure on its elastic properties, rather than study the influence of the crystalline symmetry on the latter. The visualization of such a large amount of data may by difficult; thus, let us start the examination of the influence of the inclusion by plotting the averaged values of Poisson’s ratio in the main crystallographic directions: [100], [110], and [111]. As can be seen in Figure 5a–c, any change of $\sigma^{\prime }/\sigma$ caused the average value of Poisson’s ratio to increase. This increase was particularly significant when $\sigma^{\prime }/\sigma >1$. A similar behavior was observed in systems with channels [71] and layers [72] separately. The study of Poisson’s ratio in the selected directions did not provide, however, a complete insight into the changes that occurred in the elastic properties of the system. All cubic systems of spheres ($\sigma^{\prime }/\sigma =1$), for which Poisson’s ratio was averaged with respect to all transverse directions $\langle \nu_{\left[110\right]}\rangle >0$, had specific directions for which $\nu_{[110]m}<0$ (a typical example is $\nu_{\left[110\right]\left[1\bar{1}0\right]}<0$). To better illustrate the changes in the auxetic properties induced by the change in $\sigma^{\prime }/\sigma$, one can calculate a parameter [79]:

(9) $$A=\int_{0}^{2\pi }d\varphi \int_{0}^{\pi }\sin\theta d\theta \int_{0}^{R(\theta ,\varphi )}r^{2}dr\;,$$

which may be understood as a volume of a certain space (in the spherical coordinate system) confined by the averaged negative part of the Poisson’s ratio R(θ,φ) calculated in all possible $\vec{n}$-directions. The latter can be expressed as [79]:

(10) $$R\left(\theta ,\varphi \right)=\frac{1}{2\pi }\int_{0}^{\pi }\left(\left|\nu_{n(\theta ,\varphi )}\left(\alpha \right)\right|-\nu_{n(\theta ,\varphi )}\left(\alpha \right)\right)d\alpha \;.$$

**Figure 6:**
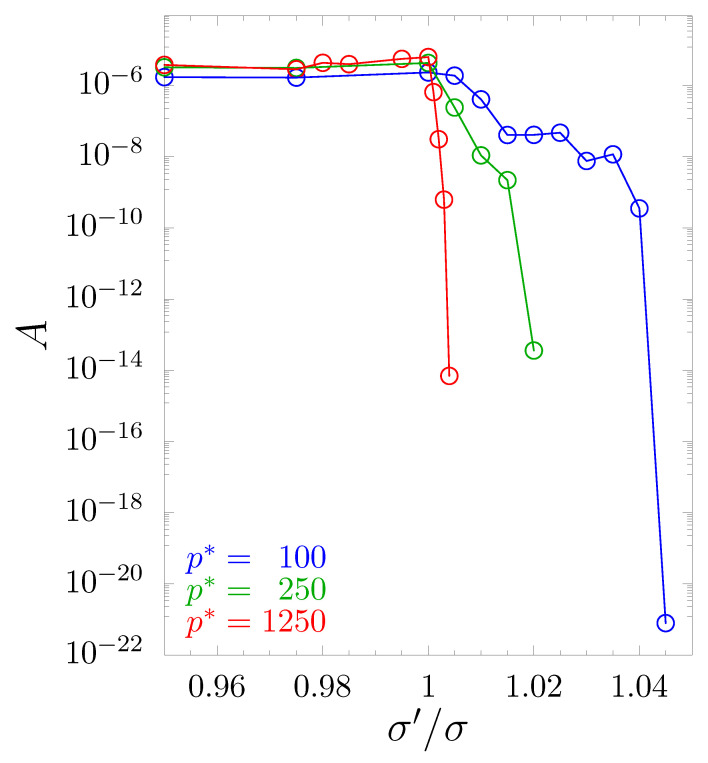
The parameter *A* (see Equation (9)), which may be understood as a volume of a certain space (in the spherical coordinate system) confined by the averaged negative part of the Poisson’s ratio in all possible $\vec{n}$-directions.

**Figure 7:**
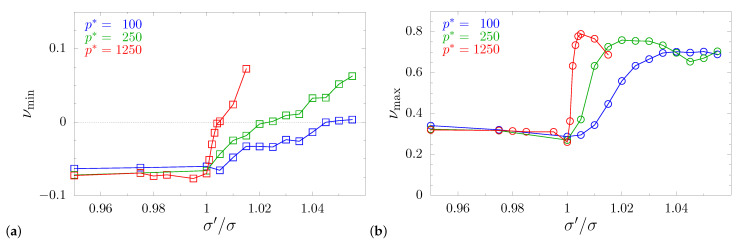
Global extreme values of Poisson’s ratio for all studied pressures plotted with respect to $\sigma^{\prime }/\sigma$. Global extremes are understood as the (**a**) minimal and (**b**) maximal Poisson’s ratio found for the system under the given pressure with given $\sigma^{\prime }/\sigma$, in any $\vec{n}$-direction. The positive sign of $\nu_{\min}$ implies that the system is non-auxetic, i.e., there is no direction $\vec{n}$ for which the system exhibits an auxetic response in any direction $\vec{m}$ transverse to it.

Figure 6 presents the numerically determined value of *A* for the studied systems, sampled in $10^{6}$ different $\vec{n}$ directions. The decreasing value *A* along with the increase of $\sigma^{\prime }/\sigma$ indicated an increase of the minimal values of Poisson’s ratio and, thus, a decreasing number of directions for which Poisson’s ratio was negative. One can observe a systematic decay of auxetic properties. The curves stopped at $\sigma^{\prime }/\sigma$ values for which there was no direction with $\nu_{nm}<0$. It was also interesting that auxeticity did not return with further increase of $\sigma^{\prime }/\sigma$ (up to the limit of the stability of the tetragonal structure). This means that the use of the inclusion completely eliminated auxeticity in the system. Thus, one obtains a model metamaterial, for which only a small modification of particle diameters was sufficient to eliminate one of the characteristic features of cubic systems [19], namely the negative value of Poisson’s ratio in $\left[110\right]\left[1\bar{1}0\right]$-direction. This effect was surprising because the hybrid inclusion was made up of components that separately had the opposite effect. The first one (nanolayer) only slightly raised the minimal Poisson’s ratio values (and only in a certain $\sigma^{\prime }/\sigma >1$ range [72]). For $\sigma^{\prime }/\sigma =1.045$, where auxetic properties were eliminated from the current model, the nanolayer alone exhibited small *enhancement* of auxeticity over the cubic system. The second (nanochannel) significantly *enhanced* auxeticity for $\sigma^{\prime }/\sigma >1.045$ [71]. However, by combining both types of inclusions, one achieves a strong increase of Poisson’s ratio, virtually in all directions. Changes of Poisson’s ratio in the system can be also observed in the plots of its global extreme values (i.e., the minimal and the maximal values observed for any of the $10^{6}$ sampled $\vec{n}$-directions) shown in Figure 7. One can see there a systematic increase of the minimum value of ν along with the increase of $\sigma^{\prime }/\sigma$ for all studied pressures.

**Figure 8:**
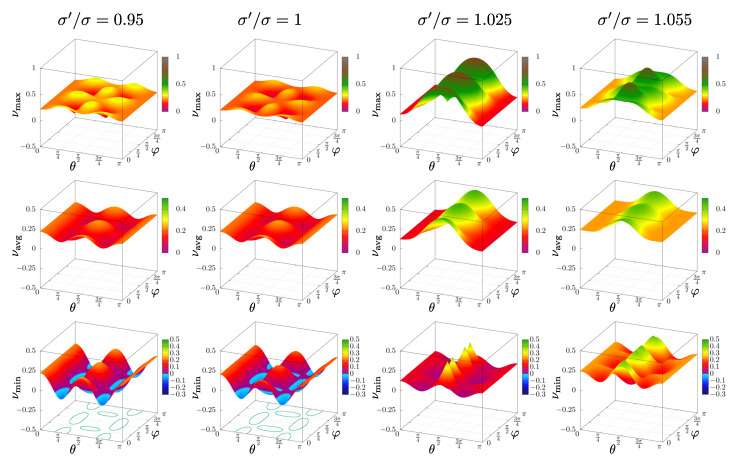
Plots of surfaces of extreme (minimal, bottom row; maximal, top row) and the average (middle row) Poisson’s ratio for given $\vec{n}$-direction, as a function of θ,φ, for $p^{*}=250$ and selected $\sigma^{\prime }/\sigma$ organized in columns from the left (I) 0.95, (II) 1, (III) 1.025, (IV) 1.055. Solid lines on the θ−φ plane are isolines for ν=0 (for θ,φ pairs inside these regions, Poisson’s ratio is negative).

To better illustrate the changes in the elastic properties of the system, one can plot not only the global extreme values, but also entire planes of minimal and maximal values of Poisson’s ratio for all $\vec{n}$-directions. Figure 8 presents (for selected values of $\sigma^{\prime }/\sigma$ and dimensionless pressure $p^{*}=250$) the surfaces of the maximal (top row), average (middle row), and minimal (bottom row) Poisson’s ratio in the range θ,φ=(0,π〉. Although every $\sigma^{\prime }/\sigma \neq 1$ resulted in a tetragonal system, one can see that the properties differed between the cases of $\sigma^{\prime }/\sigma <1$ and $\sigma^{\prime }/\sigma >1$. The former were effectively very close to the cubic systems. This was due to the fact that the matrix of particles outside the inclusion to certain extent compensated for the decreasing sizes of inclusion particles. However, in the latter case ($\sigma^{\prime }/\sigma >0$), increasing sizes of hard particles forming the inclusion significantly modified the elastic properties of the system.

**Figure 9:**
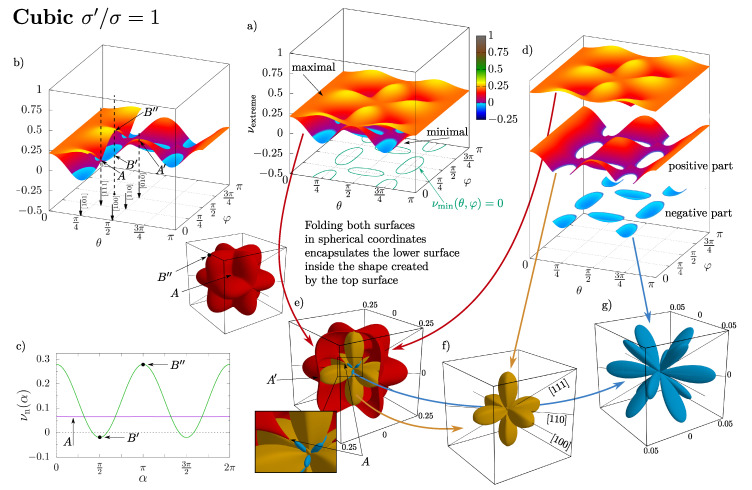

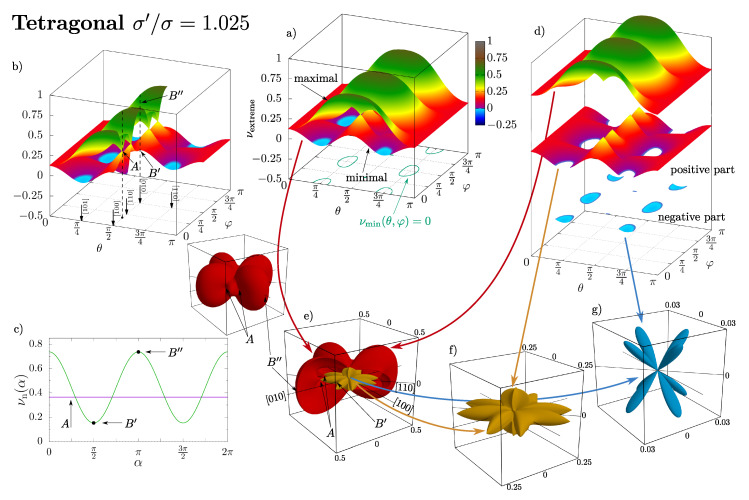
Extreme Poisson’s ratio for cubic (upper part) and tetragonal (lower part) systems. Plotted in (**a**) as two 3D surfaces of the maximal and minimal Poisson’s ratio (similar to (8)). In (**b**), the surfaces were cut in order to clearly show the internal topology and to mark the location of the typically studied crystallographic directions, as well as the characteristic points on the surfaces, e.g., isotropic Poisson’s ratio points (*A*) or extreme Poisson’s ratios for a given direction ($B^{\prime }$, $B^{\prime \prime }$). These values are also marked in (**c**) as a plot of Poisson’s ratio with respect to the orientation of the $\vec{m}$-direction (for the same $\vec{n}$-direction as marked in (**b**)). (**d**–**g**) present how the same data can be decomposed and folded in spherical coordinates resulting in the shapes presented in (**e**–**g**). The respective points from (**b**) are also marked here. For clarity, the positive and negative part of the minimal Poisson’s ratio are drawn separately (**d**,**f**,**g**).

The chart presented in Figure 9 shows the analogous surfaces, but for two selected cases at dimensionless pressure $p^{*}=100$: the cubic and tetragonal structure with $\sigma^{\prime }/\sigma$ equal to 1.025. In Figure 9b, the plotted surfaces were trimmed to show the internal topology. Typical crystallographic directions are marked for their corresponding values of θ and φ. Moreover, the selected directions for which Poisson’s ratio became isotropic (i.e., it did not depend on the $\vec{m}$-direction)—when both surfaces touched at a single point—are marked as points *A*. Particular directions that showed the strong dependence of Poisson’s ratio on the $\vec{m}$-direction are marked as points $B^{\prime }$ and $B^{\prime \prime }$, respectively, corresponding to the minimal and maximal PR value, along with the dependence of $\nu_{n}$ on the $\vec{m}$-direction (as a function of (α)), are shown in Figure 9c. Another way to visualize this data is to plot these surfaces in the spherical coordinates (Figure 9g–f). In such a case, the surface of the maximal Poisson’s ratio enclosed within itself all the remaining ones. Due to this fact and because the part ν<0 was relatively small compared to other parts, it was convenient to separate the negative part of the minimal Poisson’s ratio surface (Figure 9f,g). The shapes of the plotted surfaces and volumes that they enclosed constituted a very convenient way of showing both the symmetry of the system and its auxetic properties (see Figure 9f), or the lack of auxeticity.

## 5. Conclusions

It has been shown that the elastic properties of even such simple models as f.c.c. crystals of hard spheres may be significantly altered by small modifications of the crystalline structure. The use of an inclusion of particles, sizes of which are only a few percent greater than other particles in the system, can significantly modify elastic properties of the model material. It has been shown that, with the help of a hybrid inclusion in the form of a nanochannel joined with a nanolayer, one can completely eliminate auxetic properties from the f.c.c. crystal. It was surprising that such a small modification of the structure was enough to eliminate one of the characteristic features of f.c.c. hard sphere systems, namely the negative value of Poisson’s ratio in the $\left[110\right]\left[1\bar{1}0\right]$-direction. For all studied values of pressure, with increasing values of $\sigma^{\prime }/\sigma$, an extinction of the auxetic properties was observed. This lack of auxeticity was sustained along with the further increase of $\sigma^{\prime }/\sigma$ as long as the tetragonal system remained stable. It is worth noting that this hybrid inclusion was made from components that either slightly (nanolayer [72]) or strongly (nanochannel [71]) enhanced auxeticity.

The results presented in this manuscript showed the potential of structural modifications to significantly modify elastic properties of systems. It has been shown that controlling the sizes of nanoinclusion particles (a certain subset of the system’s particles) was an efficient method for the modification of the nanocomposites’ elastic properties. This research clearly demonstrated that not only the size modifications, but, more importantly, the shape of inclusions were of crucial importance in modifying the elastic properties. Because the HS potential is not a real interaction, but merely a convenient reference model in condensed matter [80], it rather indicates some general trends in condensed matter systems, such as, e.g., colloids, instead of describing any particular material precisely. This work pointed out some qualitative behaviors, expected in various “entropic” materials [80], that can be modeled by hard spheres. We hope that the results presented in this article will be of interest to scientists in material engineering and metamaterials.

## Data Availability

The data presented in this study are available on request from the first author (J.W.N. narojczyk@ifmpan.poznan.pl).

## Abbreviations

The following abbreviations are used in this manuscript (listed in order of occurrence in the text):

PR	Poisson’s ratio
HS	hard sphere (potential)
MC	Monte Carlo
